# A First Insight into High Prevalence of Undiagnosed Smear-Negative Pulmonary Tuberculosis in Northern Ethiopian Prisons: Implications for Greater Investment and Quality Control

**DOI:** 10.1371/journal.pone.0106869

**Published:** 2014-09-09

**Authors:** Fantahun Biadglegne, Arne C. Rodloff, Ulrich Sack

**Affiliations:** 1 Faculty of Medicine and Health Sciences, Bahir Dar University, Bahir Dar, Ethiopia; 2 Institute of Medical Microbiology and Epidemiology of Infectious Diseases, University Hospital, University of Leipzig, Leipzig, Germany; 3 Institute of Clinical Immunology, University Hospital, University of Leipzig, Leipzig, Germany; 4 Translational Centre for Regenerative Medicine (TRM)-Leipzig, University of Leipzig, Leipzig, Germany; Columbia University, College of Physicians and Surgeons, United States of America

## Abstract

**Background:**

Tuberculosis (TB) transmission in prisons poses significant risks to inmates as well as the general population. Currently, there are no data on smear-negative pulmonary TB cases in prisons and by extension no data on the impact such cases have on TB incidence. This study was designed to obtain initial data on the prevalence of smear-negative cases of TB in prisons as well as preliminary risk factor analysis for such TB cases.

**Methods:**

This cross-sectional survey was conducted in November 2013 at eight main prisons located in the state of Amhara, Ethiopia. Interviews using a structured and pretested questionnaire were done first to identify symptomatic prisoners. Three consecutive sputum samples were collected and examined using acid fast bacilli (AFB) microscopy at the point of care. All smear-negative sputum samples were taken for culture and Xpert testing. Descriptive and multivariate analysis was done using SPSS version 16.

**Results:**

Overall the prevalence of smear-negative pulmonary TB cases in the study prisons was 8% (16/200). Using multivariate analysis, a contact history to TB patients in prison, educational level, cough and night sweating were found to be predictors of TB positivity among smear-negative pulmonary TB cases (p≤ 0.05).

**Conclusions:**

In the studied prisons, high prevalence of undiagnosed TB cases using AFB microscopy was documented, which is an important public health concern that urgently needs to be addressed. Furthermore, patients with night sweating, non-productive cough, a contact history with TB patients and who are illiterate merit special attention, larger studies are warranted in the future to assess the associations more precisely. Further studies are also needed to examine TB transmission dynamics by patients with smear-negative pulmonary TB in a prison setting.

## Introduction

Tuberculosis (TB) is one of the leading causes of death in the world. Globally, around 8.8 million people develop TB and 1.45 million people die every year due to TB, of which 0.35 million deaths are associated with HIV-TB co-infection [1]. The global burden of death and disease caused by TB is concentrated especially in low-income countries. Sub-Saharan Africa, including Ethiopia, is an area with high prevalence of TB infection and in 2013; a WHO report showed that Ethiopia ranks seventh among the world's 22 countries with a high tuberculosis burden [2]. In Ethiopia, the average TB prevalence and mortality rates are 623 and 42 per 82,950 individuals respectively [1].

Prison settings have been often identified as important but neglected reservoirs for TB, including multi-drug resistant (MDR)-TB [3]; and TB in prisons threatens not only inmates, but also prison staff who eventually interact directly with their family and community when they leave work [4]. This indicates that TB in prison is not only the concern of prisoners, but concerns the wider society at large. Globally, the level of TB in prisons has been reported to be 10 to 100 times higher than that of the civilian population [5]. In Africa, TB in prisons is not well documented. However, according to the 2005 WHO report, the average TB incidence and prevalence rates of TB in sub-Saharan Africa are 363/100,000 and 475/100,000 respectively [6]. Studies in Malawi, Ivory Coast and Botswana showed higher prevalence of smear-positive pulmonary TB in prisons compared to in the civilian population [7]–[9]. This increased incidence has also been associated with the human immunodeficiency virus (HIV) epidemic [10].

In Ethiopia, limited work has been done on smear-positive pulmonary TB in prisons [11], [12]. To assess the full prevalence of TB in prison settings, the incidence of TB smear-negative cases must also be considered. Such data is currently lacking in the field. With this background, this study was designed to obtain initial data on the prevalence of smear-negative cases of TB in prisons as well as preliminary risk factor analysis for such TB cases.

## Materials and Methods

### Study design, specimen and population

**Figure 1:**
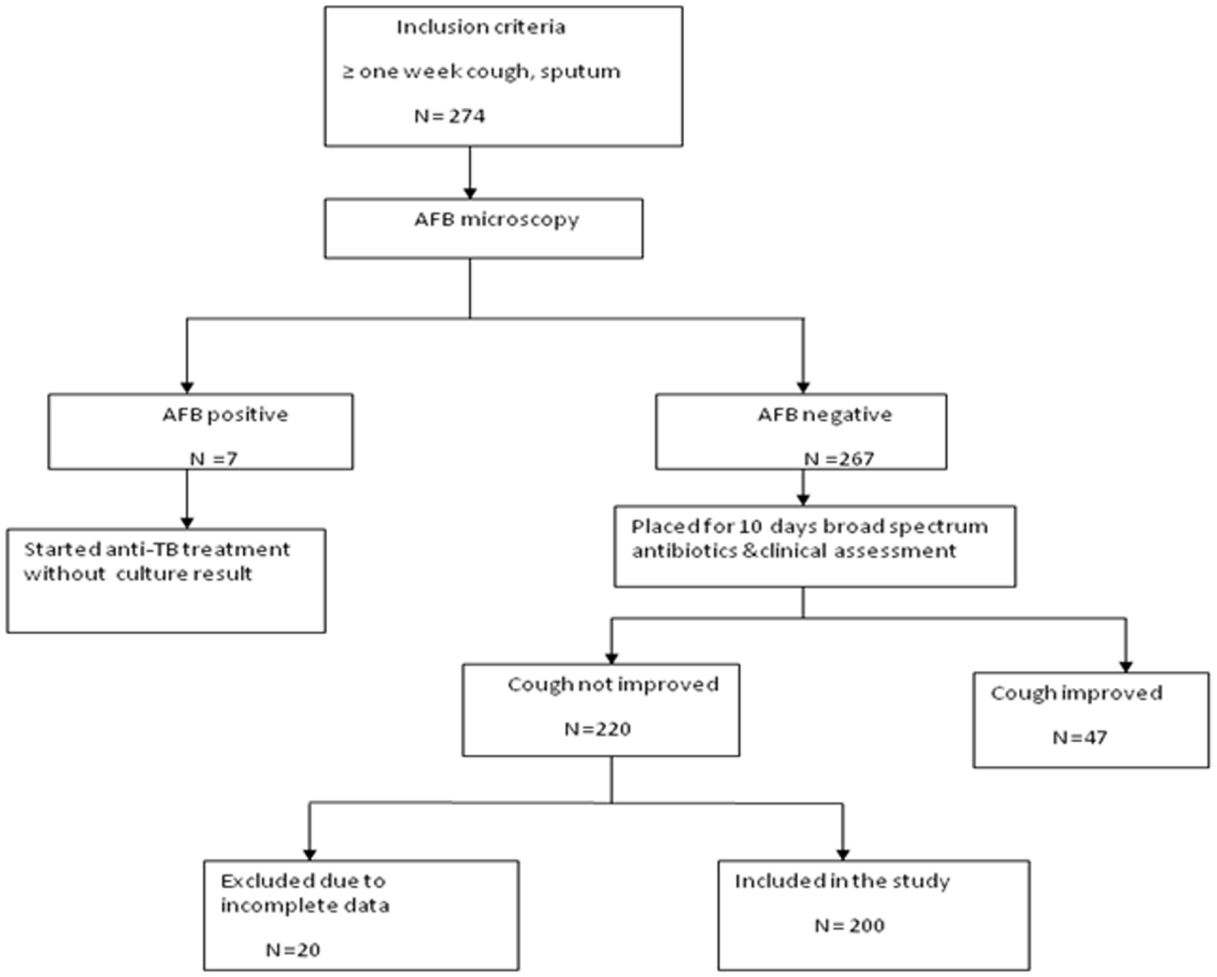
Flow diagram of the inclusion criteria for study subjects.

This survey was conducted in November 2013 at eight main prisons located in Amhara, Ethiopia. A flow diagram of inclusion and exclusion criteria for study subjects is summarized in Figure 1. Among 220 suspected smear-negative pulmonary TB patients, twenty cases were excluded due to insufficient sputum and incomplete socio-demographic data; therefore a total of 200 smear-negative cases were enrolled in this study. The diagnosis of suspected TB cases was based on national guidelines for the microscopic test of TB [13]. Spot-morning-spot sputum samples from symptomatic prisoners were collected in standardized sputum containers and all the sputum tests were done using light microscope examination of Ziehl-Neelsen (ZN) stained smears at the point of care for the presence and absence of acid fast bacilli (AFB). Prisoners with positive sputum smears were placed on anti-tuberculosis treatment as recommended by National Tuberculosis and Leprosy Control Program (NTLCP) guidelines [14], while smear-negative patients were given a 10-day course of broad spectrum antibiotic treatment. If their cough did not improve after this treatment, a second cycle of three consecutive clinical samples were collected as described above and examined for AFB. Socio-demographic information, clinical data and prison history were collected by administering a structured and pretested questionnaire. All smear-negative sputum samples were placed in sterile screw capped disposable conical tubes and stored at −80°C until transported to the laboratory in Leipzig, Germany. Specimens were packed on ice and transported according to regulations provided by the International Air Transport Association (http://www.iata.org/ads/issa/htm).

### Processing and culturing of specimens

On arrival at the mycobacteriology laboratory at the University Hospital Leipzig, all the sputum specimens were treated with the standard N-acetyl-L- cysteine (NALC)-NaOH method [15]. In brief, 10 ml of 0.5% NALC solution (4% NaOH and 2.9% sodium citrate) was added to the respective sputum sample. Then the specimens were incubated at room temperature on a shaker for 20 minutes, after which 30 ml of phosphate buffered saline (PBS) (pH 6.8) was added for neutralization and the specimens were subsequently centrifuged at 3,300×g for 20 minutes. The concentrated sputum samples were re-suspended in 1 ml of phosphate buffer and used to perform mycobacterium culture and GeneXpert MTB/RIF (Xpert) testing. Details of specimen culturing, incubation, purity check and Xpert testing for detection of MTBC and rifampicin resistance have been previously described [16], [17].

### Operational definitions

In accordance with the NTLCP guidelines [14] the following TB case classifications were applied:

- **Smear-positive pulmonary TB (PTB+) -** A patient with at least two initial sputum smear examinations positive for AFB by direct microscopy, or a patient with one initial smear examination positive for AFB by direct microscopy, or a patient with one initial smear examination positive for AFB by direct microscope and radiographic abnormalities consistent with active PTB.
- **Smear-negative pulmonary TB (PTB-) -** A patient having symptoms suggestive of TB with at least 3 initial smear examinations that were negative for AFB by direct microscopy, no response to a course of broad spectrum antibiotics, followed by an additional three negative smear examinations by direct microscopy and radiological abnormalities consistent with pulmonary tuberculosis or a patient whose diagnosis is based on a culture-positive result for *M. tuberculosis*, but has three negative initial smear examinations by direct microscopy.
- **Extra pulmonary tuberculosis (EPTB) -** TB in organs other than the lungs, proven by one culture positive specimen from an extraplumonary site or histo-pathological evidence from a biopsy.

### Statistical analysis

All data were entered, cleared, and analyzed using the SPSS statistical software package, Version 16 (SPSS Inc., Chicago, IL, USA). Descriptive data analysis was used to visualize differences within the data. Frequencies, odds ratio (ORs) and its 95% confidence interval (CI) were calculated. All covariates that were associated and non-associated with the outcome variable in the bivariate analysis were subsequently included in multivariate analysis to determine factors associated with TB positivity. A p value of ≤ 0.05 was used as the cut-off point for statistical significance.

### Ethical considerations

The study was reviewed and approved by an Institutional Review Board (IRB) of the University of Bahir Dar and the Amhara regional state prison commission, Bahir Dar, Ethiopia. After the research staff explained about TB transmission, the need for screening, the benefits of receiving treatment and prevention of TB in prison for inmates, written informed consent was obtained from each study subject. Individual records were coded and accessed only by research staff.

## Results

A total of 200 cases were enrolled in the study, out of which 195 (97.5%) were males and 5 (2.5%) were females. Their ages ranged from 13 to 73 years, with a mean age of 39 years. One hundred fifty (75%) of them were rural residents and 89 (44.5%) were found to be illiterate. Overall the prevalence of culture and/or Xpert test positive smear-negative pulmonary TB cases in the study prisons was 8% (16/200). Of the total culture- and/or Xpert test-positive, smear-negative pulmonary TB cases, culture identified 11 (5.5%) cases of smear-negative pulmonary TB and would still have missed 4 (2%) of the cases. The Xpert test identified 15 (7.5%) cases of TB and missed 1 (0.5%). Out of the total culture and/or Xpert positive cases, five cases were identified from Debiretabor, four cases from Gondar, one case from Woldia and two cases each were identified from Debremarkos, Dessie and Debirebirhan prisons. When the number of registered smear-negative pulmonary TB cases are stratified by prison setting 12 (6%), 17 (8.5%), 23 (11.5%), 24 (12%), 33(16.5%), 35 (17.5%) and 28 (14%) were from Woldia, Bahir Dar, Fenoteselam, Dessie, Deberebirhan, Debiretabor, Debremarkos and Gondar prison, respectively (Table 1).

**Table 1:** Distribution of smear-negative pulmonary TB by prison settings in Amhara state, Ethiopia, 2013, (n = 200).

Prison Setting	Culture + Xpert test + N (%)	Culture − Xpert test + N (%)	Culture + Xpert test −N (%)	Culture − Xpert test − N (%)	Total N (%)
Debrebrihan	1(3)	1(3)	0(0)	31(93.9)	33(16.5)
Dessie	2(8.3)	0(0)	0(0)	22(91.7)	24(12)
Woldia	0(0)	1(8.3)	0(0)	11(91.7)	12(6)
Gondar	2(7.1)	1(3.6)	1(3.6)	24(85.7)	28(14)
Debiretabor	4(11.4)	1(2.7)	0(0)	30(85.7)	35(17.5)
Bahir Dar	0(0)	0(0)	0(0)	17(100)	17(8.5)
Fenoteselam	0(0)	0(0)	0(0)	23(100)	23(11.5)
Debremarkos	2(7.1)	0(0)	0(0)	26(92.9)	28(14)
Total	11(5.5)	4(2)	1 (0.5)	184(92)	200(100)

+, positive,− negative, N, number.

The most prevalent systemic symptom was cough in 174 (87%) cases, followed by fever in 166 cases (83%), chest pain in 162 (81%) cases and weight loss in 160 (80%) cases. Among the study participants, 44 (22%) of them had a history of contact with TB patients in prison and 116 (58%) had received family visits during incarceration. The number of inmates per cell varies from 10–587, with the mean number of 117.4. In addition to this high population density, inmates could be held in such cells for as long as 3 years. In the multivariable logistic regression model, smear negative pulmonary TB suspects who had night sweating were more likely to have TB, with an adjusted odds ratio(AOR) of 10.09 [ 95% confidence interval (CI), 1.62–240.85]. In addition, increased TB positivity was associated with illiterate inmates [AOR: 3.79, 95%CI (1.91–15.82)] and a contact history to TB patients was associated with increased risk to present TB among the prisoners [AOR: 6.13, 95%CI (1.72–8.85)], while decreased TB positivity was associated with patients who had productive cough [AOR: 0.03, 95%CI (0.00–0.36)]. Number of inmates per cell, sex, age, fever, weight loss, chest pain, family visit, length of incarceration and presence of symptoms before imprisonment did not have association with the occurrence of TB (Table 2).

**Table 2:** Multivariable analysis of risk factors for sputum smear-negative TB among culture- and Xpert MTB/RIF test-positive cases in Amhara state prisons, 2013, (n = 200).

Variables ¥	Total	%	TB pos	%	TB neg	%	COR(95%CI)	AOR(95%CI)
Sex								
Male	195	97.5	15	7.7	180	92.3	0.33(0.04–3.17)	0.32(0.02–4.46)
Female	5	2.5	1	20	4	80	Ref	Ref
Age in years								
<30	62	31	7	11.3	55	88.7	1.82(0.65–5.15)	2.10 (0.55–8.03)
≥30	138	69	9	6.5	129	93.5	Ref	Ref
Education								
Illiterate	94	47	14	14.9	80	85.1	3.90(1.21–11.57)*	3.79 (1.91–15.82)*
Above read and write	106	53	2	1.9	104	98.1	Ref	Ref
Fever								
Yes	166	83	11	6.6	155	93.4	0.41(0.13–1.27)	1.05(0.10–11.01)
No	34	17	5	14.7	29	85.3	Ref	Ref
Weight loss								
Yes	160	80	14	8.8	146	91.2	1.82(0.39–8.36)	3.79(0.36–39.5)
No	40	20	2	5	38	95	Ref	Ref
Cough								
Productive	174	87	9	5.2	165	94.8	0.15(0.05–0.44)*	0.03(0.00–0.36) *
Non productive	26	13	7	26.9	19	73.1	Ref	Ref
Chest pain								
Yes	162	81	9	5.6	153	94.4	0.26 (0.09–0.75)*	1.08 (0.17–7.01)
No	38	19	7	18.4	31	81.6	Ref	Ref
Night sweating								
Yes	151	75.5	12	7.9	139	92.1	1.15(0.23–35.37)	10.09(1.62–240.85)*
No	49	24.5	4	8.2	45	91.8	Ref	Ref
Symptom before imprisonment								
Yes	38	19	2	5.3	36	94.7	0.58(0.13–2.70	0.51(0.07–3.89)
No	162	81	14	8.6	148	91.4	Ref	Ref
Family visit for food								
Yes	116	58	7	6	109	94	0.54(0.19–1.50)	0.66 (0.19–2.98)
No	84	42	9	10.7	75	89.3	Ref	Ref
Contact history to TB patients								
Yes	44	22	10	22.7	34	77.3	7.35(2.5–21.61)*	6.13(1.72–8.85) *
No	156	78	6	3	150	97	Ref	Ref
Length of incarceration (in months)								
≤ 12	84	42	6	7.9	78	92.1	0.80(0.28–2.29)	1.05(0.28–3.90)
>12	116	58	10	8.6	106	91.4	Ref	Ref
No. of inmates/cell								
<150	174	87	12	6.9	162	93.1	0.41 (0.12–1.38)	0.95(0.18–5.05)
≥150	26	13	4	15.4	22	84.6	Ref	Ref
Window opened?								
Yes	80	40	5	6.3	75	93.7	0.62(0.21–1.85)	0.40(0.09–1.81)
No	120	60	11	9.2	109	90.8	Ref	Ref

Ref- reference, TB pos -tuberculosis pos, TB neg- tuberculosis negative COR- Crude odds ratio, AOR - Adjusted odds ratio, CI- Confidence interval, ¥ included variables in multivariate analysis *Statistically significant, p≤ 0.05.

## Discussion

Prisons pool and facilitate the spread of smear-positive and -negative pulmonary TB, which is increasingly being reported worldwide [18]–[21]. In Ethiopia, the limited diagnostic capacity for TB in prisons remains a challenge to improving case detection rates. Our work offers the first insight into the prevalence of smear-negative pulmonary TB in Amhara state prisons, Ethiopia, and has important indications for the strategic control of TB in prisons. In this study, among 200 smear-negative pulmonary TB suspects, we found that a significant number of cases at 16(8%), were positive when subjected to culture and Xpert test. Our results are consistent with those reported by others [22], [23]. The high prevalence of smear-negative, culture- and/or Xpert test-positive cases found in this study confirms an urgent need to expand rapid, accurate and reliable diagnostic tests in prison settings in Ethiopia. However, potential differences in prevalence between smear-negative, culture positive and smear-negative, culture negative cases are possible due to several factors, including collection of unreliable sputum specimens, HIV-coinfection, duration of TB infection, the number of fields examined, the suspected cases population being examined, technical competence in sputum smearing and experiences to read smears [24], [25].

In this study all AFB microscopy smear-negative pulmonary TB cases were newly diagnosed. Thus, efforts to improve TB case detection in prison should be strengthened. A diagnostic approach using definitive diagnostic methods increased the TB detection rate and is important for diagnosis and treatment of smear-negative TB cases in this difficult setting. The results of the study revealed that the Xpert test has a better performance than culture, because it diagnosed four smear/culture-negative cases and increased the relative proportion of diagnosed smear-negative TB by 2%. However, one smear/Xpert test-negative case was diagnosed positive using culture and increased the prevalence of smear-negative TB by 0.5%, indicating the combined use of different diagnostic tests represented a significant improvement in TB detection rate among smear-negative TB cases in prison settings. Other studies have also shown that culture and Xpert test increased the rate of detection of *Mycobacterium* from smear-negative TB cases [16], [22], [23].

Frequently occurring clinical features are significant in supporting the diagnosis of smear-negative TB in areas with high TB infection [11], [18]. This study demonstrates that smear-negative inmates had similar complaints of cough, fever, weight loss, chest pain and night sweating like smear-positive cases, as was also reported earlier [26]. However, Samb et al. in 1999 [27] reported that smear-negative patients are less probable to suffer fever and weight loss than their smear-positive counterparts. Our study measured the risks associated with TB positivity among smear-negative pulmonary TB cases in the prison population. The study results showed that smear-negative pulmonary TB cases with non-productive cough and night sweating were more likely to have TB, indicating a need for prompt diagnosis and treatment of TB in prison for inmates with these symptoms to prevent the spread of TB to many other counterparts in prison [11], [18], [28]. The increased risk of TB positivity among smear-negative pulmonary TB prisoners with non-productive cough is not known. In the absence of a culture/Xpert test facility, the use of combined symptoms are key diagnostic tools for identifying smear-negative pulmonary TB, which would contribute to reducing TB in prisons by treating cases earlier [11], [18], [28], [29]. Delayed case finding, poor patient care management, lack of well-organized health services, skilled manpower or furnished laboratory facilities and lack of adequate referral systems to the nearby health institutions were major problems for prison clinics in the study sites.

In this study, the disease risk was high for illiterate inmates compared to those inmates with educational status above basic literacy (“above read and write”). One of the main explanations might be the low level of health care seeking behaviors in illiterate patients. Thus, health education for illiterate inmates is important to reduce TB transmission in prisons. A contact history to TB patients in prison was found to be a predictor of TB positivity among smear-negative TB cases, suggesting that transmission may occur in this prison population, although further study may be needed to identify transmission dynamics. Transmission could be facilitated by the nature of the cells shared by the prisoners. Our observation in the study sites showed that the cells are poorly ventilated and dark and that they contained a lot of inmates ranging from 10-587 (the mean number of inmates per cell was 117.4). The high contact rate due to overcrowding combined with poor health care management, transfer of inmates from one prison to another, from room to room and releases of inmates to community before completion of treatment without follow up increases the spread of TB and MDR-TB in prisons and in the general population [11], [12]. In this study, the period of incarceration for most of the inmates was a maximum of one year, which has been suggested as a main reason for increased TB incidence in prisons [30]. However, length of incarceration was not found to be significantly associated with TB positivity in this study, in contrast to a previous study reported elsewhere [22]. Risk factors such as age and sex may play an important role in the progression of TB infection [31]. In this study, however, there was no significantly different between the age group, males and females.

The strength of our study is that all included cases had culture and Xpert test diagnosed TB, unlike previous reports, where culture and Xpert test were not used [32], [33]. Despite this strength, our study has some drawbacks, which are subjects for future studies. (1) The lack of data on chest X-rays findings due to the lack of trained radiologists or chest physicians in the prison clinics was a deficiency in our study. It is reported that in areas of high HIV and TB prevalence, 75% of smear-negative TB cases are likely to have atypical chest radiographs [34]. Samb et al. in 1999 reported that smear-negative TB cases are less probable to have cavities on the chest radiograph. (2) The lack of data on HIV status of the patients whose sputum has processed for culture and Xpert test was another main limitation of this study. This may be important as some studies have shown that HIV infection was associated with a higher probability of smear-negative TB disease [35], [36]. (3) Another drawback of our study is that its small sample size weakened the risk factor analysis. However, the results and the study are still important in the absence of such information from any previous studies and the challenges in securing higher sample sizes in this difficult setting. Furthermore, the cross-sectional design of this study does not allow us to make definitive inferences about the effect of the risk factors in association with the outcome variables. Therefore, a longitudinal study is recommended to determine the results of this and other similar cohorts.

In conclusion, a high prevalence of undiagnosed TB cases using AFB microscopy was documented in the studied prisons, which is an important public health concern that urgently needs to be addressed. Furthermore, patients with night sweating, productive cough, a contact history to TB patients and illiterate inmates merit special attention; larger studies are needed in the future to assess the associations more precisely. In-service training of the existing technicians and regular quality control is also warranted. Further studies are required to understand TB transmission dynamics by patients with smear-negative pulmonary TB in a prison setting.
